# Expedition Cognition: A Review and Prospective of Subterranean Neuroscience With Spaceflight Applications

**DOI:** 10.3389/fnhum.2018.00407

**Published:** 2018-10-30

**Authors:** Nicolette B. Mogilever, Lucrezia Zuccarelli, Ford Burles, Giuseppe Iaria, Giacomo Strapazzon, Loredana Bessone, Emily B. J. Coffey

**Affiliations:** ^1^Montreal Neurological Institute, McGill University, Montreal, QC, Canada; ^2^Department of Medicine, University of Udine, Udine, Italy; ^3^Department of Psychology, Hotchkiss Brain Institute and Alberta Children's Hospital Research Institute, University of Calgary, Calgary, AB, Canada; ^4^Institute of Mountain Emergency Medicine, Eurac Research - Institute of Mountain Emergency Medicine, Bolzano, Italy; ^5^Directorate of Human and Robotics, Exploration, European Space Agency, Köln, Germany; ^6^Department of Psychology, Concordia University, Montreal, QC, Canada

**Keywords:** spaceflight, space analog, astronauts, neuroscience, cognition, psychology, human factors, wearable measurement

## Abstract

Renewed interest in human space exploration has highlighted the gaps in knowledge needed for successful long-duration missions outside low-Earth orbit. Although the technical challenges of such missions are being systematically overcome, many of the unknowns in predicting mission success depend on human behavior and performance, knowledge of which must be either obtained through space research or extrapolated from human experience on Earth. Particularly in human neuroscience, laboratory-based research efforts are not closely connected to real environments such as human space exploration. As caves share several of the physical and psychological challenges of spaceflight, underground expeditions have recently been developed as a spaceflight analog for astronaut training purposes, suggesting that they might also be suitable for studying aspects of behavior and cognition that cannot be fully examined under laboratory conditions. Our objective is to foster a bi-directional exchange between cognitive neuroscientists and expedition experts by (1) describing the cave environment as a worthy space analog for human research, (2) reviewing work conducted on human neuroscience and cognition within caves, (3) exploring the range of topics for which the unique environment may prove valuable as well as obstacles and limitations, (4) outlining technologies and methods appropriate for cave use, and (5) suggesting how researchers might establish contact with potential expedition collaborators. We believe that cave expeditions, as well as other sorts of expeditions, offer unique possibilities for cognitive neuroscience that will complement laboratory work and help to improve human performance and safety in operational environments, both on Earth and in space.

## Introduction

Human space exploration has been limited to orbital space flight since 1972 (Apollo 17), but due to renewed interest by traditional government entities and the private sector, this trend is about to change. Engineering challenges are being overcome that will allow for a return to the Moon, and extend exploration to deep-space asteroids and to Mars (Salotti and Heidmann, 2014; Thronson et al., 2016). However, the difficulties of future missions for which we are least prepared may be those in the human domain (Kanas and Manzey, 2008; De La Torre et al., 2012; Bishop, 2013; Sgobba et al., 2017a). Separation from family and friends, delays in communications with Earth, distortion of audio and visual signals, and limited privacy and personal space are important factors for crewmembers of long-term space missions (Sandal et al., 2006). Even the most highly selected and trained individual is subject to limitations of human physiology and psychology. The isolated, confined, extreme and otherwise unusual physical and social environments of long-duration missions will approach these limits, and potentially result in catastrophic failure (for an overview of incidents related to human error in manned space missions, see Sgobba et al., 2017a).

Risks to human health and performance can be mitigated through selection, training, mission and equipment design, and countermeasures (Kanas and Manzey, 2008), and can be investigated in a variety of ways (Bishop, 2013). The human nervous system itself is studied primarily under laboratory conditions, using neuroimaging methods such as structural and functional magnetic resonance imaging (fMRI) and magnetoencephalography (MEG) to observe the brain and the neural correlates of behavior non-invasively, and through comparisons between healthy and impaired systems by studying patient populations. It is also common to probe circuit, cellular, and molecular-level processes using animal models. While laboratory work is essential to establish a basis for interpreting field results and is generally less costly and less constrained than is research conducted in space, it also has limitations; it rarely looks at complex environments that are representative of real operational environments, and laboratory conditions cannot adequately simulate the unique conditions of spaceflight.

To better understand physiological and cognitive adaptations of the nervous system under conditions of microgravity, a series of studies using data collected in flight or pre- and post-flight has been conducted on postural reactions, eye movements, spatial orientation illusions, and cognitive responses (reviewed in Clément and Ngo-Anh, 2013). Some of the effects of microgravity on body fluid distribution (in addition to more physiological topics of bone density and muscle loss) can be simulated using bedrest studies in which the head is inclined downwards by about six degrees (a procedure that has negative consequences for mental status, Ishizaki et al., 2002), and by observing the changes in brain anatomy from pre- to post-flight (Roberts et al., 2017).

Aside from microgravity itself, the most relevant conditions of spaceflight for many other research questions about the nervous system can be found or devised on Earth. These “space analogs” may arise incidentally from other human activities, such as during Antarctic expeditions, or may be planned to simulate complex interactions of environmental, physical, physiological, and social aspects during space missions (Pagel and Choukèr, 2016). Space analogs can therefore offer platforms partway between the laboratory environment and the operational spaceflight context for the scientific study of psychology, cognition and neuroscience (Keeton et al., 2011). Neurocognitive changes, fatigue, circadian rhythm alterations, sleep problems, changes in stress hormone levels, and immune function have all been observed in situations that mimic some aspects of prospective human space missions (Pagel and Choukèr, 2016). A particularly valuable aspect of expedition-based analogs is that participants are in real, physically demanding and potentially dangerous situations with additional effects on stress, sleep, and team interactions.

In addition to informing future space mission design, space analog environments offer possibilities for neuroscientists to investigate brain function and behavioral performance in unique situations. Extending the study of human neuroscience outside the lab could lead to insights for basic research and benefits for safety-critical occupations (i.e., medical teams, shift-workers, firefighters, or air traffic controllers). However, opportunities for mutual exchange have yet to be fully exploited, likely due to limited contact between laboratory researchers and expedition experts, and because portable equipment for measuring neurophysiological signals has only recently reached a level of maturation necessary to make high quality measurements *in situ*.

Our objective here is to foster an exchange between cognitive neuroscientists, and cave expedition and space analog experts, by providing an overview of how laboratory and field research in neuroscience and related areas (i.e., cognition, cognitive psychology, neuropsychology) can be bridged, using caving expeditions as an exemplar space analog and expedition environment.

## Scope and terminology

We first discuss the properties of available space analogs and their evaluation and discuss the particular characteristics of caves that make them suitable for exerting the physical and psychological challenges of spaceflight, in order to assist researchers' selection of missions appropriate for their research questions. We review work that pertains to human neuroscience and cognition conducted to date in caves, and then explore how the few focus areas of that early work can be broadened to a range of current topics. We then outline tools and techniques that are suitable for use in cave environments. Finally, we suggest how researchers might establish contact with organizations and teams that conduct expeditions.

Neuroscience, cognitive science, neuropsychology, and psychology are broad overlapping fields that may each study the same or related processes with numerous tools. We will not attempt to distinguish between the purviews of these fields here; research questions from any domain that concern environment-brain-behavior relationships that affect human performance are our focus. These topics at times overlap with human physiology, human factors, sports psychology, and social psychology. Although it makes little sense to study human performance in isolation from other physiological processes and from a physical and social context, other resources exist that have dealt specifically with these topics. For references on medical and physiological matters (i.e., bone loss, radiation, extravehicular activities, balance, motion sickness and nutrition), and for information on physiological and neurophysiological studies conduced to date on the ISS (see Buckey, 2006; Clément and Ngo-Anh, 2013). For space flight human factors research methods, accident analysis and prevention, and human-automation interaction (see Sgobba et al., 2017b; Kanki, 2018b; Marquez et al., 2018; Wilson, 2018). Psychology, mental heath, team performance and group interactions in space are reviewed in (Suedfeld and Steel, 2000; Manzey, 2004; Kanas and Manzey, 2008; Kanas, 2015; Salas et al., 2015; Pagel and Choukèr, 2016; Sandal, 2018). For a discussion of current knowledge on neuroplastic changes in the human central nervous system associated with spaceflight (actual or simulated) as measured by magnetic resonance imaging-based techniques (see Van Ombergen et al., 2017). Cognitive functions, human error, and workload and fatigue are relevant to expedition cognition and are amenable to study in the cave environment as discussed here; useful references for further reading include (De La Torre et al., 2012; Gore, 2018; Kanki, 2018a).

## Space analogs and assessment of suitability

The National Aeronautics and Space Administration (NASA), European Space Agency (ESA), Roscosmos State Corporation for Space Activities, Canadian Space Agency (CSA), and other space exploration organizations have created a variety of terrestrial and aquatic space analogs, as well as simulated missions. Each analog simulates a subset of space or extra-terrestrial conditions. Those analogs which are predominantly used to test equipment, validate procedures, and gain an understanding of system-wide technical and communication challenges emphasize the equivalence of physical factors, such as terrain, reduced gravity and communications delays; those with natural sciences foci might emphasize geological and biological properties of the analog (e.g., the yearly NASA/ESA–funded Arctic Mars Analog Svalbard Expedition in Norway is used for testing astrobiological hypotheses).

Other analogs have a human focus or mixed scientific uses including human research. For the purposes of human activities, the relevant conditions for a particular topic of interest may include additional factors that affect a crew member's ability to carry out their work efficiently and safely. An important principle for assessing the relevance of various extreme environments as viable analogs for space or providing the basis for cross-comparison is that it is the *experience* of the environments rather than the environments themselves that must be considered (Suedfield, 1991; Bishop, 2013). Thus, an environment may provide an excellent analog for spaceflight without physically resembling it, provided that many of the stressors exerted upon human participants are paralleled. For example, as in space, the external environment in the Antarctic winter requires specialized equipment, planning, and procedures in order to safely conduct operations outside the habitat. Morphew enumerated the stressors of (long-duration) spaceflight (see Table 1; Morphew, 2001).

**Table 1:** Stressors of long duration space flights (Morphew, 2001).

**Physiological/Physical**	**Psychological**	**Psychosocial**	**Human Factors**	**Habitability**
Radiation	***Isolation and confinement***	***High team coordination demands***	***High and low levels of workload***	***Limited hygiene***
***Absence of natural time parameters***	***Limited possibility for abort/rescue***	Interpersonal tension between crew/ground ***(not common, but possible with some team configurations)***	***Limited exchange of info/comms with external environment***	***Chronic exposure to*** vibration and ***noise***
***Altered circadian rhythms***	***High-risk conditions and potential loss of life***	Family life disruption	***Limited equipment, facilities and supplies***	***Limited sleep facilities***
***Decrease in exposure to sunlight***	***System and mission complexity***	***Enforced interpersonal contact***	***Mission danger and risk associated with: equipment failure, malfunction, damange***	***Lighting and illumination***
Adaptation to microgravity	Hostile environment ***(possible in some caves)***	***Crew factors (i.e., gender, size, personality, etc.)***	Adaptation to the artificially engineered environment ***(some parallels to unusual natural environment)***	***Lack of privacy***
***Sensory/perceptual deprivation of varied natural sources***	***Alterations in sensory stimuli***	***Multicultural issues***	***Food restrictions/limitations***	Isolation from support systems ***(likely limited effect due to mission durations)***
***Sleep disturbance***	***Disruptions in sleep***	“Host-Guest” phenomenon	Technology-interface challenges ***(possible with some missions)***	
Space Adaptation Sickness (SAS)	***Limited habitability (e.g., limited hygiene)***	***Social conflict***	***Use of equiment in microgravity conditions (not present, but some parallels in 3D cave environment)***	

*Stressors that may be paralleled in cave environments are highlighted (bold, italics)*.

In Antarctica, McMurdo Antarctic Research Station (population > 1,000) is used by NASA as a Mars analog because of terrain, temperature, and taxing conditions comparable to those of Mars' surface (Morris and Holt, 1983). Psychiatric studies at McMurdo station have provided evidence that prolonged isolation can increase the risk for mental health disorders (Kanas, 2015). ESA collaborates with the smaller Franco-Italian Antarctic base Concordia (population~15) (Tafforin, 2009), at which some human research is conducted, for example on sleep quality and adaptation to high altitude conditions (Tellez et al., 2014). Although aquatic environments are not precise models for the physical conditions of asteroid, moon, or planetary exploration, underwater missions do mimic the stressors associated with safety, communication, and technological logistics related to long-term spaceflight and exploration. The NASA Extreme Environment Mission Operations (NEEMO) is an underwater research lab where crews are sent on missions up to 2 weeks long to focus on testing equipment and procedures for future spacewalks (Todd and Reagan, 2004), and the Pavillion Lake Research Project (PLRP; CSA/NASA) uses remotely operated, autonomous, and human explorers to investigate microbiology and remnants of early life.

Simulated missions provide a similar physical environment to a spacecraft or base habitat, as well as activities and schedules resembling those of astronauts. One of the most ambitious of such projects in recent history (2007, 2011) was Mars500 (ESA/Russian Institute for Biomedical Problems). In the longer of two experiments, six volunteers were confined in a mock-up spacecraft for over a year and a half in order to simulate a complete Mars mission. Mars500 included a number of experiments on human brain function and behavior whose results have been published. Research topics included the effect of exercise on prefrontal cortex activity (Schneider et al., 2013); circadian heart rate variability during isolation (Vigo et al., 2013); and the relationships between cortisol levels on brain activity, sleep architecture, and emotional states (Gemignani et al., 2014); sleeping patterns (Basner et al., 2013); and the relationship between feelings of loneliness and cognitive functions (Van Baarsen et al., 2012). Other recent/ongoing projects are exploring perception of time, sleep quality, concentration, and their biological clocks over periods of weeks (Lunares, Poland), and crew selection, team processes, self-guided stress management and resilience training, crew communications and autonomous behavioral countermeasures for spaceflight in missions of several months (Hawaii Space Exploration Analog and Simulation; HI-SEAS; NASA/University of Hawaii).

Training courses that are designed as space analogs have also been proposed as suitable environments in which to conduct human research. ESA's Cooperative Adventure for Valuing and Exercising human behavior and performance Skills (CAVES) program, in which astronauts conduct scientific and exploration tasks in subterranean environments, is one such possibility (Strapazzon et al., 2014). NASA uses the National Outdoor Leadership School (NOLS) to tests the ability of astronauts and candidates to work together in a challenging outdoor setting (Alexander, 2016). For more information about space analogs, please refer to Keeton et al. (2011), Lia Schlacht et al. (2016), Pagel and Choukèr (2016), and Kanki (2018b).

In order to categorize the wide variety of earth-space analogs, NASA created an Analog Assessment Tool (described in NASA/TP−2011-216146, Keeton et al., 2011) that helps investigators select an analog based on study goals. Initially, the tool arranges the analogs based on importance weightings where the research characteristics (such as team size or degree of physical isolation) and utility characteristics (such as relevance of the crew's tasks or the cost of the study) are proposed. Fidelity weightings are calculated for each proposed analog based on the research and utility characteristics including the degree of their isolation, hostility, confinement, risk, prior knowledge (the accessibility of information about the environment that the mission crew has access to prior to expedition), natural lighting, logistics difficulty, remote communications, science opportunity, similarity to planet surface, and sensitivity (susceptibility to damage by humans of the environment). Both sets of weightings are combined to produce an overall ranking for all proposed analogs according to the goals of the mission (Keeton et al., 2011). ESA has also analyzed facilities that are suitable to be used as lunar analogs (Hoppenbrouwers, 2016). Table 2 presents a synthesis of the criteria commonly used to evaluate terrestrial space analogs against a research project's goals.

**Table 2:** Summary of criteria for evaluating terrestrial space analogs.

**Criterion**	**Examples and notes**
Isolation/Confinement	High isolation in underwater/extreme environments like desert/Antarctica/caves
Risk	Underground, underwater, and polar missions pose more risk due to climate and proximity of medical facilities
Prior knowledge (magnitude to which information about the environment is available to crewmembers before their mission)	Artificially simulated missions are easier to predict than underwater analogs. Underground missions can offer a unique combination of known/unknown
Natural lighting	Research-topic dependent, e.g., perceptual errors, circadian rhythm, etc.
Logistics difficulty (measure of resources needed to constantly supply crewmembers)	Land-based missions like those taking place in the desert provide easier re-supplying than underwater and underground missions such as NEEMO/CAVES
Remote communications (capability to exchange information with crewmembers not physically taking part in the analog mission)	Land-based missions like those done in Antarctica or in the desert provide easier communication than underwater and underground missions such as NEEMO/CAVES
Similarity to planet surface	Desert and underground missions simulate the appearance of the Martian surface
Sensitivity (susceptibility of environment to disruption by human-activities)	Underwater and underground missions and polar regions may be sensitive to ecosystem disruption

## Caves as space analogs

Approximately 20% of Earth's landmass is karstic, i.e., consisting of topography formed from the dissolution of soluble rocks such as limestone, dolomite, and gypsum, and characterized by sinks, ravines, caves, and underground streams (Ford and Williams, 2007). Only a small portion has been explored, but many sites attract people for recreational and scientific purposes. It is estimated that at least 2,000,000 people in the US alone visit caves each year (Hooker and Shalit, 2000) and members of national speleological societies (e.g., approximately 10,000 members in the US National Speleological Society and about 7,000 in the French Federation of Speleology, gleaned from their websites) suggest that the number of people likely to be involved in rigorous expeditions worldwide is in the range of tens of thousands. Caves are, in fact, interesting to a variety of scientific disciplines, including geology, hydrogeology, and biology, but they also represent unusual challenges for the people who work, explore, rescue, and temporarily live within them. The majority of deaths of cave explorers are caused by falls related to human error, followed by rock falls, drowning, and hypothermia (Stella-Watts et al., 2012; Stella et al., 2015). Science conducted on cave expeditions therefore has the potential to significantly increase research to the benefit of spacefarers, and to improve safety in a widely practiced activity.

Caves have been identified as a naturalistic space analog for training purposes (Bessone et al., 2013; Strapazzon et al., 2014; Pagel and Choukèr, 2016). As space analogs, caves feature many logistic challenges and stressors (e.g., isolation and confinement, risk and reliance on technical equipment for safety, limited prior knowledge of the environment, unusual lighting and sensory conditions, communication and supply difficulties). The spaceflight stressors highlighted in Table 1 (in bold, italics) indicate those spaceflight stressors which are frequently present in caves conditions. Although speleological expeditions may vary in their coverage according to mission, team, and environmental properties, strong overlap is observed. Critically, cave expeditions (as well as some aquatic and polar analogs) fulfill the important psychological factor of being somewhat risky and safety-critical environments in which participants are reliant on equipment and teammates, with limited and slow rescue options (Stella-Watts et al., 2012; Bessone et al., 2013). Perceived risk is likely to cause neurophysical changes that affect many aspects of brain and behavior, from interpersonal interactions to sleep and cognitive function (Pagel and Choukèr, 2016). Cave exploration also requires discipline, teamwork, technical skills and a great deal of behavioral adaptation (Bessone et al., 2013). Martian caves and lava tubes have been proposed as suitable locations in which to construct habitats on Mars, due to thermal stability and shielding from radiation and micrometeorites (Moses and Bushnell, 2016), which would further increase the similarity of the model's physical environment.

For these reasons, the European Space Agency (ESA) has carried out training activities in the subterranean environment since 2008. The multidisciplinary mission known as CAVES is used for training astronauts of the International Space Station (ISS) Partner Space Agencies (USA, Russia, Japan, Canada, and Europe) (Bessone et al., 2013; Strapazzon et al., 2014). During the 6-day mission, astronauts conduct exploration and scientific activities under similar scheduling and mission conditions as they will later experience in space as a means of eliciting and coaching behavioral competences (Bessone et al., 2008). The science program includes environmental and air circulation monitoring, mineralogy, microbiology, chemical composition of waters, and search for life forms adapted to the cavern environment, and increasingly, human experiments.

As CAVES participants are highly selected astronauts-in-training whose objectives are to explore and conduct scientific studies, it lies toward the higher-fidelity end of the spectrum of cave analog possibilities, and of possible experimental control. However, its capacity to support multiple experiments is limited by tight personnel scheduling. Expeditions of other organizations may therefore be more suitable for a given research question, taking into consideration the specific expedition's space analog suitability (for recent examples of cave-based human research and a description of the cave conditions and mission, see Stenner et al., 2007; Antoni et al., 2017; Pinna et al., 2017). Cave expeditions may vary due to differences in cave environments (temperature, presence of water, remoteness and access, difficulty level, etc.), mission (duration, objectives, group size, group composition), organization (scientific, exploration, amateur), and the demographics of participants (age, sex, training, culture, language). These factors affect the nature of the data collection that is possible as well as its quality and applicability to other groups. In the section entitled “Connection to in-field study experts and cave community” we list some of the main speleological meetings and organizations through which expeditions appropriate to a research program might be found.

## A brief history of early neuroscientific work conducted in caves

Health outcomes of humans living in isolation have been studied over the last 80 years. In the 1960s, researchers began to investigate how biological rhythms were affected when living underground, without “zeitgebers” (i.e., environmental cues that can alter the internal clock, the study of which is now included in the field of chronobiology). Early studies involving isolation in subterranean conditions are listed in Table 3, along with their findings. These studies, as well as those later studies found in Table 4, were identified by a literature search of life science electronic databases (Medline: 1966-Present, NASA Technical Reports Server: 1915-Present, Google Scholar: Present, Worldcat: 1971-Present, OPAC: 1831-Present, and PubMed: 1997-Present). Search terms included “cave/s,” “cave” AND “isolation” AND “human,” “free-running isolation,” “potholing/ers,” “caving,” “social isolation,” ‘subterranean” AND “isolation,” “spelunking,” and “underground environment.” Because many early studies were only reported in their original language, we additionally searched for Italian: “grotta/e,” “isolamento in grotto,” “isolamento spazio temporale,” and “Montalbini” (author); French: “grotte,” “sejours souterrain,” “Siffre” (author); and Spanish: “cueva,” “aisolamento in cueva,” “permanecer bajo tierra,” and “spelunka.” All studies reporting results from human subjects in subterranean environments with a neuroscience or cognitive component were retained (16 reports).

**Table 3:** Subterranean studies reported from 1938 to 1974.

**Study**	**Subject(s)**	**Days in isolation**	**Main findings**
1. Kleitman 1938 (reported in Wolf-Meyer, 2013)	2 adult males (together; Kleitman and Richardson)	32	The goal was to change the circadian sleep-wake rhythm to a 6 day week (6 days of 28 h). One subject was able to achieve this 28 h sleep-wake rhythm but the other subject had trouble doing so and kept his initial 24 h sleep-wake rhythm
2. Mills, 1964	1 adult male (Workman)	105	Subject went to sleep and awoke later each day (~24.5 h clock); potassium excretion followed a similar cycle
3. Aschoff, 1965	1 adult male	10	Subject exhibited very unstable sleep-wake rhythm & urinal excretion rhythm but eventually stabilized at ~25.9 h
4. Halberg, 1965 (Comptes Rendus de l'Académie des Science)	1 adult male (Siffre)	62	The heart rate and sleep-wake cycles shifted to about 24.6 h; significant desynchronization of circadian sleep-wake rhythm was evident
5. Reinberg et al., 1966	1 adult female (Laurens)	88	The sleep-wake rhythm became slightly lengthened (24.5 h); menstrual cycle was shortened (by 3 days); core temperature cycles remained unchanged with respect to pre-isolation baseline
6. Siffre et al., 1966; Ghata et al., 1969, see also discussion in Halberg et al., 1970	2 adults (1 male, 1 female; Senny and Laurens), separately isolated	Male: 125 Female: 88	Temporary modifications of the visual functions, mainly on the speed of the chromatic vision were seen (pre-post isolation testing); circadian rhythms in urinary excretion and rectal temperatures were maintained but sleep-wake cycles were slightly delayed to 24.5 h
7. Fraisse et al., 1968	2 adult males (Siffre and Mairetet), separately isolated	Male (Siffre): 58 Male (Mairetet–note that different aspects of this experiment were reported in several studies): 174	The sleep-wake circadian rhythm was slightly extended (~24.5 h) for Siffre; Mairetet developed circabidian (48 h) sleep-wake rhythm; subjects' estimation of short time intervals (i.e., counting to 60 s) were the same as time estimations prior to isolation but subjects' estimation of longer time intervals (i.e., how many hours had passed since waking up and eating lunch or dinner) was underestimated by ~45%
8. Colin et al., 1968	1 adult male (Mairetet)	174	Rectal temperature period fluctuated between 18 and 31 h but eventually stabilized at a 24.5 h rhythm during the last 4 months; sleep-wake cycle was unstable throughout the whole experiment ranging from 24 to 46 h rhythms
9. Apfelbaum, 1969 (La Presse Medicale)	7 adult females (all together in isolation but slept in 2 tents)	14	People sharing the same tent had the same rhythm; a sleep-wake circadian rhythm was maintained for both groups, but still was extended in duration (~24.7 h)
10. Oléron et al., 1970	1 adult male (Mairetet)	174	Time estimation (counting to 60 s) accelerated and reaction time increased in isolation; a circabidian (48 h) sleep-wake rhythm developed
11. Mills et al., 1974	1 individual adult male (Lafferty)	127	Activity habits approximated a period of 25.1 h whereas urinary electrolyte excretion indicated a shorter period, of 24.6 h

**Table 4:** Subterranean studies reported from 1974 to 1994.

**Study**	**Subjects**	**Number of days in isolation**	**Main findings**	**Neurophysiological measures**
1. Chouvet et al., 1974	3 adult males (Mairetet, Chabert and Engelender), separately isolated	2 males in 1968 (Chabert and Engelender): 150 1 male in 1966 (Mairetet) 174	Subjects developed a circabidian/bicircadian rhythm (34 h of wakefulness and 14 h of sleep); the duration of sleep stages 3 and 4 was correlated with the duration of the previous waking period, providing evidence of homeostatic regulation mechanisms in sleep regulation	EEG, EMG, EOG
2. Siffre, 1987 (Proceedings of a Colloquium on “Space & Sea”)	Review: 7 adults from previous studies, separately isolated	60–174	5 subjects developed a circabidian/bicircadian rhythm; REM sleep duration is directly proportional to the duration of sleep (same subjects as in Chouvet et al., 1974); REM and SWS increased at the cost of lighter sleep stages (1 and 2) when the sleep wake cycle desynchronizes from circadian to circabidian	EEG, EMG, EOG
3. Sanchez da la Pena et al., 1989 (Proceedings. Second Annual IEEE Symposium on Computer-based Medical Systems)	1 adult female (Follini)	97	Subject maintained circadian systolic, diastolic, and mean arterial pressure rhythms that were slightly but significantly greater than 24 h. A circaseptan rhythm for heart rate was observed	Heart rate monitor only
4. Sonnenfeld et al., 1992	1 adult female	131	Sleep-wake cycle began to deviate from 24 h after 30 days of isolation, and thereafter ranged from 25 to 42 h in cyclical patterns	None reported
5. Hillman et al., 1994a,b (New Trends in Experimental and Clinical Psychiatry)	1 adult female (Le Guen)	103	Subject maintained a circadian sleep-wake rhythm but it varied slightly throughout the period of isolation, to a period somewhat longer than 24 h. Other physiological measurements such as urinary water excretion rate and caffeine metabolism developed circasemiseptan (half-weekly) rhythms (the authors attributed these rhythms to exposure to cosmic rays)	None reported

*Note that “circabidian” refers to 2-day (~48 h) rhythms, whereas “bicircadian” refers to twice-daily (~12 h) cycles*.

Reports from the early to mid 1900s on the effects of isolation on the human body are limited in their sample size and lack standardized methodology (Halberg et al., 1970). One of the first peer-reviewed studies to examine chronobiology was performed by Mills, who analyzed chronobiological aspects of his subject throughout 105 days in subterranean isolation (Mills, 1964). From the 1960s to the mid-1970s, similar studies documented renal rhythms, sleep-wakefulness cycles, time estimation, internal temperature, heart rate, and even menstrual cycles of their subjects as biomarkers for changes of their internal clocks (see Table 3). The majority of these earlier studies using basic physiological measures found that a rest-activity cycle persisted in the absence of any environmental synchronizer or deliberate scheduling, although it appeared to be slightly desynchronized/longer than 24 h (~24.5 h). These findings were interpreted as evidence that the internal clock does not need external cues such as intense light to regulate its biological rhythm (Halberg, 1965). Social cues (e.g., subjects sleeping in the same underground conditions nearby one another, subjects eating meals together) were also shown to affect circadian rhythm, as those of subjects isolated together tended to align (Apfelbaum, 1969).

Although some of the studies in Table 3 were able to look at physiological parameters such as the effects of isolation on vision, measures were mostly implemented prior to and after isolation as opposed to within the cave environment itself. During the expedition, circadian rhythms were observed using core body temperature, sleep-wake cycles, and subjective estimation of time.

Electroencephalography (EEG), electromyography (EMG), and electrooculography (EOG) are techniques that are used to record electrical activity in the brain, skeletal muscles, and eye movements, respectively. Due to advances in electrophysiological tools in the mid 1970s, it became possible to make physiological and neurophysiological measurements during subterranean isolation studies. One of the first cave research studies using EEG and EMG was performed by Chouvet et al. (1974), who characterized sleep architecture during isolation (i.e., the pattern of rapid eye-movement or REM sleep; light sleep or stages 1 and 2; and deep or slow-wave sleep, SWS, that occurs over a nights' rest). From the mid-1970s to the 1990s, similar studies documented the effects of isolation with limited external time cues on circadian rhythms using EEG, EMG, and EOG, in addition to the previously mentioned physiological measures. These studies are listed in Table 4, along with their main findings.

The studies presented in Tables 3, 4 represent pioneering efforts investigating circadian rhythms in the absence of an externally imposed day-night cycle. Early observations that humans have endogenous rhythmicity in biological processes and alertness levels that can be modified by external cues stimulated further research on human circadian rhythms and sleep cycles which have grown into fields of scientific study with implications for health and disease (Kirsch, 2011). Many of these studies are noteworthy for their pioneering efforts, ingenuity of design, and commitment of their subjects; isolating individuals for long periods would now be considered highly unusual (if not unethical; although causality certainly cannot be inferred, one of the subjects isolated alone for 3 months later died by suicide Hillman et al., 1994b). However, today the data generated by these studies are primarily of interest for historical reasons; the very small sample sizes and lack of experimental control and methodological standardization between studies limit the interpretability and generalizability of the findings, and the tools and practices of measurement of human psychology and physiology have evolved considerably in the interim. Later work showed that some of the findings reported above were likely caused by the experimental procedures. Most notably, many studies in Tables 3, 4 suggested that the endogenous human circadian cycle is closer to 25 than 24 h. This was later attributed to phase shift due to exposure to bright artificial light that subjects were allowed to use while awake; in the absence of bright light, the intrinsic pacemaker is in fact very close to 24 h (Czeisler et al., 1999).

## Research topics: opportunities, considerations, and collaborations

**Figure 1:**
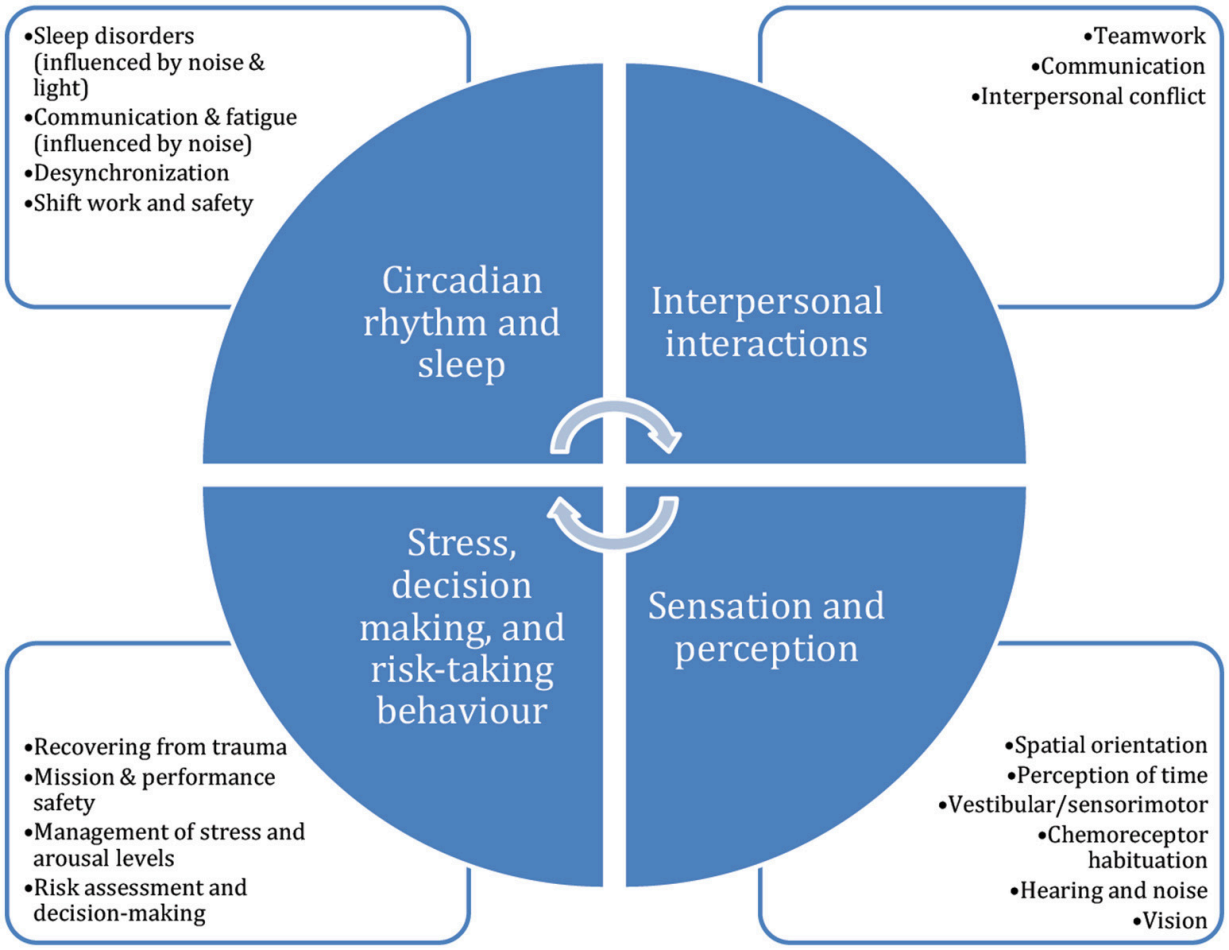
Potential topics in psychology, cognition, and neuroscience that could benefit from study in subterranean and expedition environments. Caves could also be a useful context within which to evaluate and optimize the effects of equipment interfaces and operational protocols on human cognition and performance, as well as within which to test the effectiveness of countermeasures.

Studies in caves to date have only concerned themselves with a few of the topics for which the cave environment makes a good space analog (i.e., isolation, lighting). Figure 1 presents some of the (interrelated) topics within neuroscience, cognition, and psychology that could be usefully studied in cave expeditions, and might benefit from an intermediate research platform between the laboratory environment and space itself.

### Circadian rhythm and sleep

Sleep quantity and quality, circadian rhythm, and resulting alertness levels and performance proficiency are often altered in spaceflight for environmental and operational reasons (Mallis and DeRoshia, 2005). Due to logistic challenges with sleep measurements in the spaceflight environment, only a few astronauts have been studied using polysomnography (PSG), the gold-standard method for evaluating sleep. According to astronauts' subjective reports and objective recordings of neurophysiology (i.e., EEG, PSG) and of activity levels (i.e., actigraphy; wrist-worn accelerometers) human sleep has been reported to be shorter and shallower during various missions including Skylab missions (Frost et al., 1975, 1976), space shuttle missions (Monk et al., 1998; Dijk et al., 2001), Mir missions (Gundel et al., 1997), and ISS Expeditions from 2006 to 2011 (Barger et al., 2014), compared to sleep on the ground. Barger and colleagues additionally found that the use of sleep-promoting drugs, which are known to alter sleep architecture and cognitive performance, were pervasive during spaceflight; the authors argued for the need to develop effective countermeasures to restore normal sleep in space (Barger et al., 2014).

The degree to which spaceflight sleep problems are caused by altered physiology due to the effects of microgravity itself or other factors such as isolation and confinement, noise, changes in physical activity, long or unusual sleep-wake and crew shift-work schedule, over-excitation, demographics, rapid succession of light and dark exposure, and ambient temperature is not yet known (Gundel et al., 1997; Pandi-Perumal and Gonfalone, 2016). However, results from space analogs also report significant changes in sleep patterns during Antarctic overwintering (Steinach et al., 2016) and during extended confined isolation (Basner et al., 2013), respectively, suggesting that sleep disturbance can be usefully studied in space analog conditions. In caves, mission-like levels of activity and scheduling, psychological pressures relating to factors such as risk and interpersonal interaction, new surroundings, temperature, humidity, and noise can all affect sleep timing, duration, and quality. Cave expedition constraints can also introduce circadian disturbances; for example, it is not uncommon for cave exploration activities to involve extended periods of wakefulness and near-continuous activity (>24 h and even up to 40 h), when sleeping within the cave is logistically difficult or impossible. These extended periods of wakefulness parallel those in spaceflight which can occur for operational requirements for example rendezvous and docking procedures, and in emergencies. Some cave expeditions may last weeks and require crew to adapt to sleeping and working conditions, providing a situation analogous to longer mission phases. Even when a normal sleep-wake cycle is maintained, important zeitgebers are absent or altered in caves, as in space exploration.

Inadequate sleep can affect daytime alertness levels, response time, vigilant attention, and error rates, learning, complex task performance, emotional evaluation, risk assessment, and fatigue; however, effects differ according to the type of task and degree of its complexity, characteristics of the individual, and the nature of the sleep deprivation (i.e., acute deprivation, or chronic restriction; Wickens et al., 2015; Bermudez et al., 2016; Havekes and Abel, 2017; Krause et al., 2017). In meta-analyses, mental fatigue was shown to also have some effect on physical and athletic performance (Van Cutsem et al., 2017; McMorris et al., 2018), which has relevance for more physically strenuous expedition activities such as climbing or extravehicular activities. Hypnotics (i.e., drugs used to treat insomnia) reduce sleep latency and increase sleep duration, but the resulting sleep shows abnormal sleep architecture (Cojocaru et al., 2017) and does not entirely restore impaired cognitive performance (Verster et al., 2016). Because sleep architecture is important to learning and memory consolidation (Diekelmann and Born, 2010; Ros et al., 2010), these effects are especially undesirable wherever learning is required, as it is during exploration and spatial navigation. People are not always good at assessing their own performance levels; devising means of assessing readiness to perform safety-critical tasks is important, as is knowing how well self-reported alertness levels accurately reflect subsequent cognitive performance (Boardman et al., 2017), and how well performance can be improved in the short term. Caffeine can mitigate some of the next-day cognitive performance effects of reduced sleep in a somewhat predictable fashion (Ramakrishnan et al., 2015). Interestingly, an individual's performance impairments due to sleep restriction, or enhancement due to stimulants like caffeine, may not translate directly into group performance impairments and improvements, due to mediating factors of group dynamics (Faber et al., 2017), which would also be useful processes to understand under expedition conditions.

In addition to inadequate sleep quality and duration, sleep timing affects performance. Recent progress on the molecular-genetic basis of circadian rhythms indicates that they affect cognition, learning and memory, mood, and metabolism directly, in addition to indirectly through their influence on sleep (Kyriacou and Hastings, 2010). The effect of sleep restriction and circadian misalignment is a topic of concern in occupations that involve shift-work like emergency medicine, in which short-term cognitive deficits have been related to shift work schedules (Machi et al., 2012).

### Sensation and perception

Caves offer unusual sensory inputs that affect waking behavioral performance. In caves as in enclosed artificial environments such as spacecraft, olfactory input is monotonous sometimes negative (i.e., body odors), contributing to habitability and comfort issues. Noise is pervasive in artificial environments, and is known to cause annoyance, disturb sleep and daytime sleepiness, and to negatively affect patient outcomes and staff performance (in hospitals), increase the occurrence of hypertension and cardiovascular disease, and impair cognitive performance (Stansfeld and Matheson, 2003; Basner et al., 2014a). Operational limits on both continuous and intermittent noise exposure have been established for spaceflight, in order to provide an acceptable environment for voice communications and for restful sleep (Allen et al., 2018). However, recent evidence suggests that even low levels of noise, within the established limits, can cause neurophysiological changes that negatively affect health, learning, and memory performance (studied in rodents Cheng et al., 2011). In humans, noise increases the cognitive load associated with understanding speech and communicating, and the ability to do more than one task simultaneously (Rönnberg et al., 2010). Many cave environments have continuous background noise from wind and water movement that could be used to study its effect on individual stress levels, concentration, cognitive performance, fatigue, workload, communication, and interpersonal interaction.

Lighting in caves is produced by headlamps, which create partial, focal illumination of complex three-dimensional spaces and complicates movement and navigation. These perceptual conditions are likely to increase cognitive load and contribute to dual-task performance decrements, including communication and teamwork. The type and distribution of lighting on the exterior of spacecraft affects human visual performance and is an important factor in spacecraft design, particularly for extravehicular activities (Rajulu, 2018). Future extra-terrestrial cave/lava tube exploration may create related challenges.

Higher-level perceptual skills are also relevant for spaceflight. Visuo-spatial orientation skills refer to the ability of individuals to make use of information available in the environment to efficiently orient and navigate. This function relies on cognitive processes such as memory, attention, perception, mental imagery, and decision-making skills (Ekstrom and Isham, 2017). It allows individuals to become familiar with the environment and to integrate information about self-position and orientation into a spatial mental representation of the surroundings, known as a cognitive map (Tolman, 1948; Arnold et al., 2013). Cognitive maps allow any target location from anywhere within the environment to be reached, even by following novel routes when a known pathway is unavailable (Epstein et al., 2017). An accurate mental representation of the environment is crucial for a variety of cognitive tasks in near-space, such as those that involve reaching and grasping objects from a given location within the environment or directing attention to elements in space that are not necessarily within our focal vision. These skills are necessary for maneuvering safely in microgravity, during extra-vehicular activities, and for exploration of planet surfaces (Clément and Reschke, 2008).

The ability to form accurate mental representations of the environment implies the integrity of a complex extended network in the brain (Ekstrom and Isham, 2017; Ekstrom et al., 2017). Within this network, regions in the medial temporal lobe (i.e., hippocampus and parahippocampal cortex) are involved in the learning and memory aspects of orienting and navigating through the environment (Epstein et al., 2005; Iaria et al., 2007). Interestingly, these networks are among those implicated in sleep-related processes of memory consolidation, notably of memory involving spatial and contextual elements (Diekelmann and Born, 2010). Other brain regions used while moving throughout the environment and locating elements within it include the posterior parietal cortex, which is critical for integrating different sensory information processed through our visual, vestibular, somatosensory, and proprioceptive systems (Posner et al., 1984; Andersen, 1997); and the frontal and prefrontal cortex which are necessary for executive functions such as planning, mental imagery, and working memory (Owen et al., 1990; Petrides and Baddeley, 1996). Recent studies have shown that even a minimal functional alteration (not damage *per se*) of the neural networks described above is associated with impairments of spatial processing (He et al., 2007; Iaria et al., 2014; Kim et al., 2015). As with many complex skills, maintaining expertise in spatial orientation and navigation also requires consistent practice; reliance on GPS technology for example, which offloads the cognitive demands of navigation, is associated with lower navigational expertise (Ishikawa et al., 2008) and lower hippocampal volume and connectivity (Maguire et al., 2000; Iaria et al., 2014). Spatial orientation and navigation are a clear example of a cognitive process in which one must “use it or lose it” (Shors et al., 2012).

### Stress, decision-making, and risk-taking behavior

Factors affecting physiological and psychological well-being like increased social isolation, confinement, altered sleep, and higher stress levels are also known to affect cognitive skills. For example, visuo-spatial orientation and its neural correlates (Glasauer and Mittelstaedt, 1998; Stranahan et al., 2006; Lukavský, 2014; Valera et al., 2016). Poor quality sleep leads to slower performance and more errors navigating a newly-learned environment (Valera et al., 2016), and chronic stress is known to produce spatial orientation deficits (Mizoguchi et al., 2000; Kleen et al., 2006), likely by perturbing the neurochemistry of supporting networks (Conrad, 2008, 2010; Li et al., 2015). Spatial confinement may also have more direct effects on spatial orientation, for instance, Lukavský (2014) identified a marked difference in scene memory in the six participants of the Mars500 project. Relative to controls, these individuals developed a greater bias toward “boundary extension” while viewing distant scenes, i.e., falsely recalling a wider field of view or more distant perspective from these visual stimuli. Lukavský hypothesized that the lack of interaction with distal objects and scenes due to extended stays in a relatively confined environment will result in the deterioration of the perception and strategy use within larger environments.

The hippocampus and prefrontal cortex have a well-documented sensitivity to some of the negative factors associated with subterranean explorations. Rodents housed in confined, isolated, or simple environments have smaller hippocampi, comprised of fewer neurons (Kempermann et al., 1997) with fewer dendritic spines (Leggio et al., 2005), less neurogenesis (Olson et al., 2006), and poorer spatial abilities (Nilsson et al., 1999; Leggio et al., 2005). While the typical experiences of a lab rodent differ from that of an average human, these findings are generally supported by human research (Gianaros et al., 2007; Lupien et al., 2007; Ganzel et al., 2008; Prince and Abel, 2013). The prefrontal cortex is vulnerable to both acute and chronic stressors, with acute stress producing notable impairments in spatial working memory (Arnsten, 2009), as well as reducing the capacity to problem-solve and think flexibly. Paralleling the effects seen in the hippocampus, long-term exposure to stressors produces lower prefrontal cortex volumes (Cerqueira et al., 2007), reduced dendrite length and branching (Holmes and Wellman, 2009), and detriments to spatial memory (Cerqueira et al., 2007; Arnsten, 2009), vulnerabilities that appear to worsen with aging (McEwen and Morrison, 2013). Cave environments offer challenging three-dimensional environments in which to move and explore, simulating the challenging perceptual and mission conditions of spaceflight; they may also offer unique opportunities to contribute to knowledge of hippocampal function, dysfunction, and plasticity as it relates to sleep, stress, and confinement.

The perceived risk and danger aspect of expedition environments offers another set of research opportunities with spaceflight relevance. Communication with the outside world may be very limited. Though teams often set up a telephone line between an external base and a main cave base camp for extended expeditions, difficult terrain may still require hours or even days of movement before communication can be established, and rescue attempts could take much longer. In future long-duration space missions to Mars and for permanent stays on the Martian surface, transmissions between ground and space may be delayed up to 40 min or even blocked, and short-term rescue may be impossible; lack of a visual link to Earth will add to the feelings of isolation and autonomy (Horneck and Comet, 2006; Strapazzon et al., 2014). Under uncertain conditions, stress impacts decision-making and risk-taking behavior (reviewed in Morgado et al., 2015). These effects appear to be mediated by stress-related release of neurotransmitters that lead to alterations in neural firing, and if stress is chronic, to architectural changes in frontal lobe areas involved in higher-level cognition (Arnsten, 2015). The stress associated with risk and danger also affects interpersonal interactions and group dynamics, potentially leading to feedback cycles in communication that foment crew conflict (Kalish et al., 2015).

### Interpersonal interactions and teamwork

The interaction of stressors that challenge cave and space explorers with interpersonal dynamics is a critical component of mission success (Bishop et al., 1999; Sandal, 2018). Although teamwork, team cohesion, team effectiveness, and resilience have been identified as knowledge gaps and are current topics of investigation for space exploration, there have been relatively few studies in extreme environments and space-analogs (for a summary, see Salas et al., 2015; Sandal, 2018), and studies within caves are scarce (for examples, see Bishop et al., 1999; MacNeil and Brcic, 2017).

The empirical study of team characteristics and processes has non-standard, evolving theoretical constructs and methodology (Cronin et al., 2011; Alliger et al., 2015; Kozlowski, 2015). Common techniques include behavioral observation (during simulations or training; in person, or by reviewing recordings) and self-report by surveys (during pauses in activity, or retroactively) (Brannick et al., 1997). These methods may require an uninvolved observer, rely on team members' ability and desire for introspection, and may not capture how the team dynamically reacts and interacts to changing situations. Wearable physiological and neurophysiological measurement devices have been proposed as a means of unobtrusively tracking team dynamics, assessing the quality of teams' performance in real time, and adaptively rearranging team or task components (Stevens et al., 2011; Salas et al., 2015; Santoro et al., 2015; Lederman et al., 2017). These promising approaches are in early development phases, and could be tested in cave environments.

### Evaluating interfaces and countermeasures

As well as observing and characterizing the behavioral and neurophysiological correlates of environmental stressors (Alonso et al., 2015), (neuro) physiological indices of attention, workload, and emotional state can be used to measure how people interact with technology, for the purposes of evaluating equipment interfaces (Liapis et al., 2015) and to validate brain-computer interface (BCI) systems. Passive BCIs use these signals to adapt the behavior and functionality of highly complex and safety critical systems accordingly to the user's actual mental state in real time, without requiring effort. They are promising means of optimizing interaction with technology for spaceflight applications as well as in various Earth-based applications (Coffey et al., 2010; Aricò et al., 2016; Arico et al., 2017). In cave exploration, interaction and supervision of swarms of robotic agents is a possible application (Fink et al., 2015; Kolling et al., 2016).

The cave environment could be used to test the feasibility and effectiveness of countermeasures. In a recent meta-analysis, mindfulness-based meditation was shown to reduce stress, depression, anxiety and distress, and improve quality of life in healthy individuals (Khoury et al., 2015). Neurofeedback, in which users are given a visual or auditory representation of certain features of their brain's ongoing activity such that they can learn to modulate it (e.g., based on the amplitude of different frequency bands measured with EEG), might be tested as a means of maintaining function during expeditions. In a review of about 30 controlled studies, EEG-neurofeedback showed evidence of performance gains on sustained attention, orienting and executive attention, memory, spatial rotation, reaction time, complex psychomotor skills, implicit procedural memory, recognition memory, perceptual binding, intelligence, mood and well-being (Gruzelier, 2014).

Slow oscillations present in deep sleep can be enhanced using a method known as auditory closed-loop stimulation. Short bursts of quiet broadband noise are played to the user, precisely timed to the ascending phase of ongoing slow oscillations (Ngo et al., 2013). The brain's reaction to the sounds strengthens the slow oscillations and improves some types of memory (i.e., hippocampus-dependent declarative memory; Arnal et al., 2017; Besedovsky et al., 2017). Another new method of enhancing learning is known as targeted memory reactivation (TMR), in which an olfactory or auditory stimulus is associated with a learning event. In the subsequent sleep period, the stimulus is repeated, presumably reactivating the memory and increasing the strength with which it is consolidated (learned) (Schouten et al., 2017). Although these methods are new and have shown improvements on only basic tasks that are far removed from those performed in the operational environment, further developments may make them usable to optimize learning in expedition environments; these could be tested in caves.

Thus, as early twentieth century researchers deduced, cave environments are useful for studying sleep and circadian processes. Though early studies only took advantage of the isolation and the absence of zeitgebers found in caves, a much larger set of questions can be asked during modern expeditions: of sleep and circadian rhythm, but also about sensation and perception, spatial navigation, interpersonal interactions and teamwork, human factors design, stress, and the impact of these stressors on wellbeing and performance. In the following section, we discuss several considerations for conducting human research in cave environments.

## Methodological considerations for conducting research in caves

Because of the long planning time for many space and analog missions and because of the difference in the scale of research investment, the pace of progress is generally more rapid in mainstream neuroscience. The focus of analog research is likely to be establishing and characterizing phenomena under expedition conditions and assessing the effect of interventions, whereas a laboratory approach can investigate finer-grained mechanisms, in tightly controlled paradigms that isolate specific phenomena, possibly using highly specialized equipment. Both are valuable; to maximize the advantages of each, researchers might choose to include a lab-based control group for comparison, test subjects before an expedition in the lab to serve as a baseline, complement field studies with investigations of the same phenomenon using their full lab suite, or carefully validate field equipment and procedures against laboratory standards, according to the research question.

Scientists only familiar with the traditional academic research side may find the collection *Space Safety and Human Performance* Sgobba et al. (2018), as well as Clément and Ngo-Anh (2013) to be useful starting points to review studies conducted in space or space analogs to date. It can be helpful to obtain first- or second-hand knowledge of the cave expedition environment prior to planning experiments, such that environmental and mission constraints that could introduce problems, confounds, or poor quality data can be avoided. In field studies, in fact, we often have poor control over confounding environmental factors (Brugger et al., 2018), but choosing the right “cave setting” can offer a certain level of standardization.

Expedition or medical experts who might wish to add neuroscience questions to their programs may discover too late that their results are unpublishable. For example, in auditory cognitive neuroscience, it is considered essential to confirm that subjects have normal hearing thresholds such that experimental findings can be attributed to some condition of interest and not a hearing deficit. Norms and best practices such as this have evolved in each specialized sub-field in order to guard against artifacts and confounds, and ensure replicability and generalizability of findings, but may not be obvious to operations personnel. There are often means of satisfying such requirements, once they are known. In this example, the researcher could conduct a basic audiogram on-site or arrange (with the subject's permission) to obtain equivalent information via previous medical reports. A more problematic issue concerns sample size and statistical validity of the proposed research design; (i) case studies or very small sample sizes are unlikely to be well regarded by many peers in neuroscience; (ii) small sample sizes (~*n* < 30) can show only large differences and many aspects may remain hidden due to the low power (type II error or high false negative rate). Possible solutions include collecting more extensive data on a few more homogeneous sample subjects, using different converging methods of neuroimaging and behavioral testing, pooling data over several missions (documenting differences between the missions that might affect results), or contrasting results with a laboratory-base group.

Researchers may run into differences in expectations for scientific communication when crossing field boundaries. Most space and analog human research work is currently presented in space or applied physiology-themed journals and conferences (with some exceptions of recent publications in generalist journals, e.g., Basner et al., 2013; Antoni et al., 2017). People working on related issues from the expedition and laboratory sides are therefore unlikely to be present at the same meetings or to read the same reports. Publishing in generalist journals may help to increase communication between space and mainstream neuroscience. Both specialized scientific knowledge and expedition expertise is needed to successfully take advantage of this unique situation while ensuring the value of results to the different communities.

## Tools and equipment for expedition cognition

High quality, lightweight, relatively inexpensive devices to measure (and even influence) brain activity are becoming available, as are inexpensive laptops and tablets that can be used to measure a variety of important aspects of cognition and behavior. In psychology and neuroscience (as in other fields), there is a movement toward “open science,” in which research, data, and tools are made freely available. These developments will make much more extensive expedition cognition research possible.

**Figure 2:**
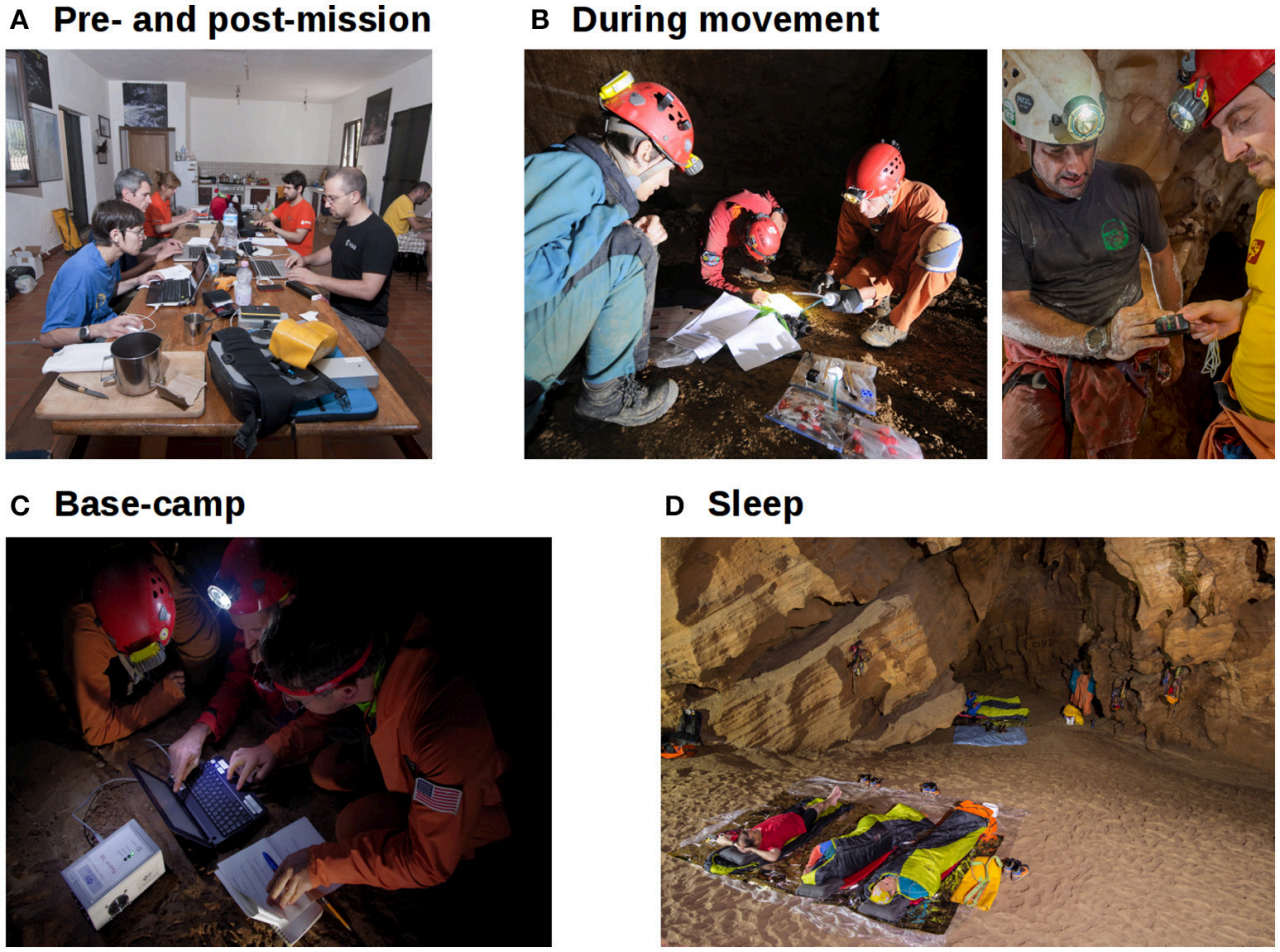
Expedition phases determine the nature of possible measurements. **(A)** Pre- and post-mission, testing can include delicate equipment and can be conducted in comfort. **(B)** During exploration, minimal portable equipment can be worn continually or used during brief stops (i.e., during photography, mapping, and rest stops); simplicity of use and robustness are key. **(C)** Where base-camps are established, more elaborate testing with laptops and electrophysiology can be conducted, as well as for sleep recordings during rest periods **(D)**. Permission has been obtained from the individuals for the publication of these images. Photo credits, ESA archives, used with permission from photographers; **(A)**, Alessio Romeo; **(B)**, left: Alessio Romeo, right: Natalino Russo; **(C)**, Vittorio Crobu; **(D)** Riccardo DeLuca.

**Figure 3:**
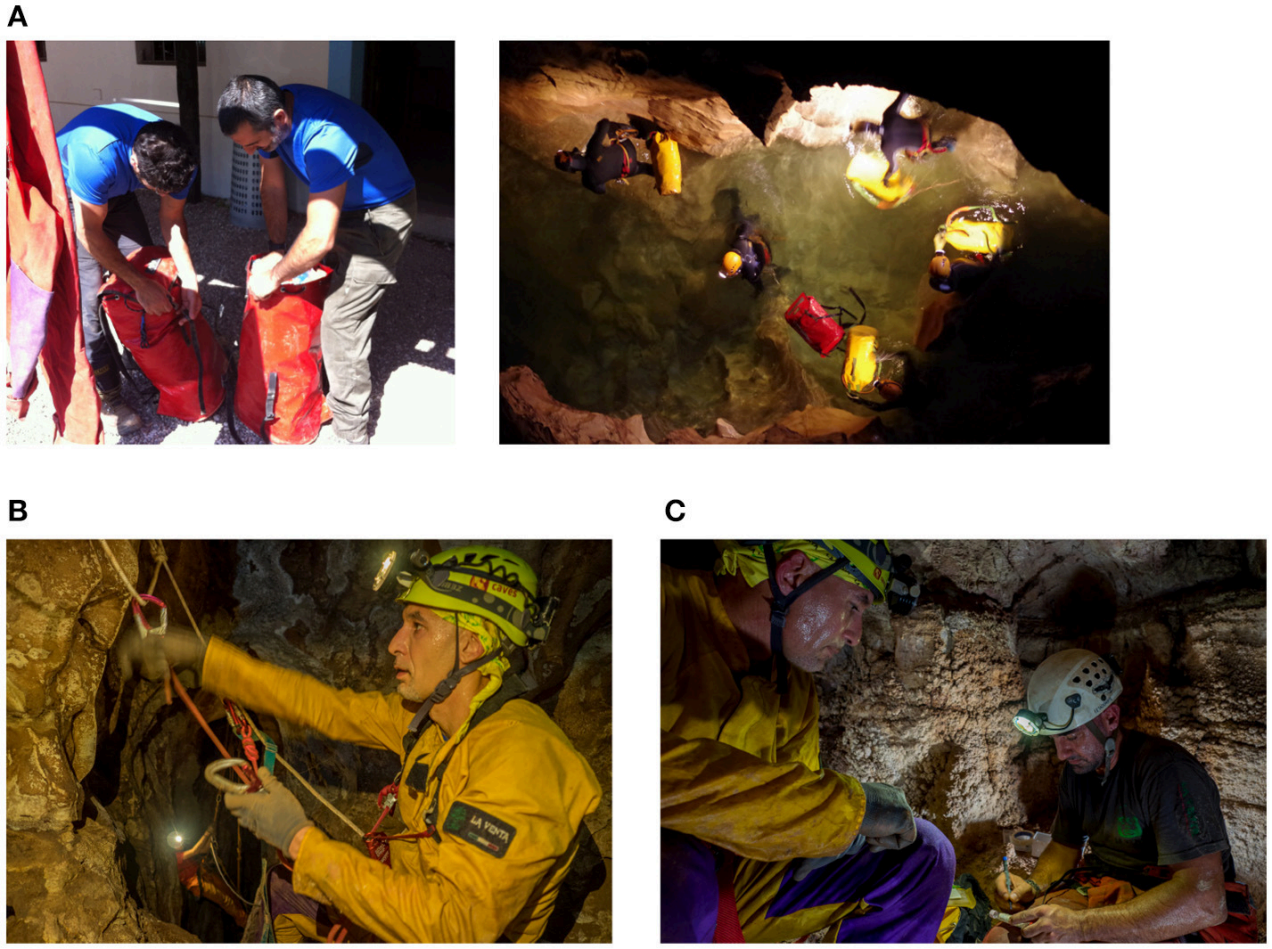
Experimental and equipment considerations. **(A)** Equipment must be small, lightweight, well-organized, and packed to protect it against damage according to the nature of the cave and expedition. **(B)** Equipment worn during movement must be positioned so as not to pose safety risks (e.g., no obstruction of view or dangling wires), not to be dislodged or damaged by climbing harnesses and activities, and so as to be protected from impact and water damage. **(C)** Consideration must be given to the conditions under which measures are administered; compliance and data quality may be affected by participant comfort. Permission has been obtained from the individuals for the publication of these images. Photo credits, ESA archives, used with permission from photographers; **(A)**, left: Loredana Bessone, right: Vittorio Crobu; **(B)**, Natalino Russo; **(C)**, Natalino Russo.

The determining factors for planning subterranean studies are the specific characteristics of the cave and mission (i.e., cave conditions, duration, group demographics, planned activities, logistic restrictions), and the part of the expedition during which measurements need to take place (illustrated in Figure 2). Factors other than cave and mission itself should also be considered when selecting a research situation, such as the presence of external scientific, logistical, and medical resources, and presence of an organized rescue organization. Of the mission phases, pre-post expedition periods are the least constrained, as data could be measured external to the cave in a portable lab or nearby research facility. In-cave equipment is transported in water-resistant bags on shoulders, pushed and dragged through constricted areas, and raised or lowered on ropes. It must be small, light, and protected against high humidity, submersion in water in some caves/passages, dust, mud, and shock due to handling (illustrated in Figure 3). Power consumption may be a limiting factor of equipment, especially for longer missions; electronics should be as small and energy-efficient as possible. Pencils and moisture-resistant paper for simple measures like surveys might be more appropriate than electronic solutions when power is a constraint, although even water-resistant paper is easily soaked and soiled, and pencils, lost. Measurements taken in the cave at the end of the day at a base camp or during the rest period for sleep studies could allow for relatively larger or more delicate equipment or equipment that requires some setup to be used, like laptops and EEG.

Researchers should keep in mind that their experiments will be secondary to expeditioners' other goals; time-consuming or irritating procedures, unclear instructions, and measurement equipment failures requiring troubleshooting may reduce compliance and decrease data quality or increase dropout to a greater extent than in the laboratory. For extended expeditions, thought should be given as to how to maintain comfort and good signal quality despite limited opportunities for personal hygiene. The main tools currently available for human neuroscience in caves, ordered by increasing degree of complexity are discussed in the following subsections.

### Surveys and questionnaires

Surveys and questionnaires are common research tools, particularly toward the psychological end of the psychology-neuroscience spectrum where they are used to answer questions about subjective experience, obtain reports of habits or schedules, or document interpersonal interactions. When cognitive or neurophysiological measures are the main tools, surveys and questionnaires can also be useful to gather information about health and demographic variables (e.g., to rule out neurological disorders or to document the subject's age and gender), and about potential confounds that may be necessary to interpret the data correctly. Methods to develop effective surveys and questionnaires are described in Yorubaland et al. (1999) and Fink (2003). A variety of prepared and validated surveys and questionnaires are available, many of which can be obtained free of charge and have been validated. Existing measures may already have been linked to other neurophysiological data, allowing for some further insight and inference; for example, daily ratings of positive mood have been linked to serotonergic function in the central nervous system (Flory et al., 2004). However, scales are often developed for clinical or diagnostic use and might not be sensitive to minor variations between healthy, high functioning adults, or a scale may be intended to give a global general score such as propensity toward daytime sleepiness rather than measure daily fluctuations. Some tools are lengthy or require a trained interviewer to administer, which would not be appropriate in an expedition setting. A further consideration is the questionnaire load of the participants and the conditions under which the participant will complete the survey (e.g., physical comfort, lighting), which can affect data quality. Nonetheless, surveys and scales can be useful to quickly and inexpensively collect useful data, and require no equipment other than pen and (water-resistant) paper, or they can be computerized or answers can be recorded by voice according to mission constraints.

We list some common questionnaire-based tools in Table 5 which fall into several topic categories. The unusual sensory conditions and need for alertness due to risks required in expedition environments can contribute to fatigue. Alertness, sleepiness, and fatigue levels are interrelated but distinct phenomena that can be measured with questionnaires, in addition to physiological measures such as pupillometry (described below). Situational awareness is the perception and understanding of the environment and events, and is particularly relevant to operational tasks. Stress is a state of mental or emotional strain or tension resulting from adverse or demanding circumstances; space flight and cave expeditions both pose stressors on team members. Prolonged missions in isolation and under stressful conditions can affect the mood and therefore the capacity for mission members to perform to the best of their abilities (Liu et al., 2016). Positive team dynamics and social interactions are highly desirable in long-term missions, during the taxing conditions of space-flight, and the longest cave expeditions, which can be weeks long. Pre-mission questionnaires that examine social compatibility have been used to improve group interactions in isolation experiments (Dunlap, 1965; Chidester et al., 1991). These questionnaires can be useful in crew selection (see Kanas et al., 2013).

**Table 5:** Questionnaire-based tools.

**Topic area**	**Questionnaire**	**Description**	**Application in space medicine or related examples**	**Notes about perspectives in cave analogs**
Alertness, sleepiness, and fatigue	Stanford Sleepiness Scale (Hoddes et al., 1973)	A self-reported 7 point scale which assesses how fatigued or low-functioning an individual may feel on a daily basis	Used to study sleep efficiency in relation to neurobehavioural performance for space operations (Mollicone et al., 2008) and in the Mars expedition analog experiment at the Mars Desert Research Station in Utah (Groemer et al., 2010)	Useful for long cave expedition (>3 days) to observe interactions with sleep and circadian rhythm factors, expedition activities, interpersonal interactions, and teamwork
Alertness, sleepiness, and fatigue	The ZOGIM-A Alertness Questionnaire (Shahid et al., 2011)	A 10 item scale that examines subjects' daily energy levels (taking into account caffeine consumption and exercise levels) and quantifies the amount of alert-demanding tasks performed over the course of that day	Proven to be reliable in measuring alertness levels (Moller et al., 2006; Shapiro et al., 2006)	As above
Alertness, sleepiness, and fatigue	Toronto Hospital Alertness Test (Shapiro et al., 2006)	A retrospective questionnaire that assesses the subjects' perception of their alertness levels, daily	Proven to be reliable in measuring alertness levels (Moller et al., 2006; Shapiro et al., 2006)	As above
Situational awareness	Situational Awareness Rating Technique (SART) (Taylor, 1990)	Implemented after another task and asks the subject to rate their own awareness and performance on a 7-point scale	Designed for aircrew systems testing and human factors studies	Could be used after various mission-related activities
Situational awareness	The Situation Awareness Global Assessment Technique (SAGAT) (Endsley, 1995)	Implemented by randomly freezing an operational simulated situation so that subjects can immediately answer questions about performance and awareness	Used on astronauts operating a planetary rover (Fong et al., 2014)	Could be integrated with computerized cognitive testing, or non-safety-critical operational tasks
Stress	The Perceived Stress Scale (PSS) (Cohen et al., 1983)	A widely used general questionnaire designed to measure the degree to which situations in one's life are appraised as stressful	Used in cognitive neuroscience for between-group control purposes (e.g., Maguire et al., 2006)	Appropriate for long-term evaluation, for example to establish level of perceived stress upon study entry between individuals or to establish equivalency of stress levels between groups
Mood and emotion	The Beck Depression Inventory (Beck et al., 1961)	A 21-item questionnaire used to assess the intensity of depression by asking the subject to rate articles such as pessimism, crying, agitation, or loss of interest, on a 4-point scale - either on a daily, weekly, or monthly basis	Used in prolonged isolation studies such as the Mars520 study (Basner et al., 2014b)	Appropriate for long-term evaluation, for example to track levels of depression before, during, and after extended cave permanences
Mood and emotion	Positive and Negative Affect Schedule (PANAS) (Watson et al., 1988)	A pair of 10-item self-report scales evaluating the extent individuals experience particular feelings or emotions, as rated on a 5-point scale	Used to study crew members on a 2 week mission at the Mars Desert Research Station (Sawyer et al., 2012), and during the Mars105 space simulation (Nicolas et al., 2013)	As above
Mood and emotion	The UWIST Mood Adjective checklist (UMACL) (Matthews et al., 1990)	Subjects judge the magnitude of the moods they experience weekly on a 5 point scale	Used in the Mars520 space simulation to study the psychological adaptations of crew members (Polackova Solcova et al., 2014)	Can be administered before another task to assess interactions of mood and performance
Mood and emotion	Profile of Mood States (POMS) questionnaire (McNair et al., 1971)	Subjects rate 65 items within 7 mood domains (anger-hostility, vigor-activity, confusion-bewilderment, depression-dejection, tension-anxiety, and friendliness) on a 5 point scale	Used to study ISS astronauts' moods over time and their relation to scores of fatigue, anger, hostility and depression (Kanas and Manzey, 2008)	Appropriate for long-term evaluation, for example to track mood states during, and after extended cave permanences; the fatigue-inertia scale may be particularly relevant for sleep
Mood and emotion	Core Self-Evaluations Scale (CSES) (Judge and Erez, 2003)	Subjects indicate their agreement with 12 statements (e.g., “I complete tasks successfully”) on a 5-point scale. Used to rate individuals' overall self-worth and capability, as well as four dimensions (i.e., locus of control, generalized self-efficacy, self-esteem, and emotional stability)	Used widely to study job satisfaction and job performance	Appropriate for pre-mission testing, to compare individuals and groups, potentially interesting to study how individual differences relate to performance under extended mission conditions
Mood and emotion	New General Self-Efficacy scale (NGSE) (Chen et al., 2001)	An 8-item scale that measures subjects' general belief that they have the capacity to complete a task successfully, using a 5-point scale	Used widely in studies of team efficiency, and performance	As above
Mood and emotion	The trait of emotional stability can be assessed using the 60-item neuroticism scale from the International Personality Item Pool (Goldberg, 1999)	Scale items require individuals to rate the accuracy of a statement about them (e.g. “Am often in a bad mood”), on a 5-point Likert scale	Used widely in studies of team interactions, leadership, and performance	As above
Teamwork and social dynamics	System for Multiple Level Observation of Groups (SYMLOG) (Keyton and Wall, 1989)	An interpersonal rating method for analyzing interaction among group participants. Subjects rate the frequency with with 26 behaviors occur in relation to other group members, on a 3-point scale.	Proven sensitive to decreased crew cohesion that lead to social isolation of several crew members in the ESA isolation study ISEMSI (Isolation Study for European Manned Space Infrastructures), and in the MIR space station simulation (Sandal et al., 1995; Sandal, 2001)	Appropriate for periodic evaluation of group interactions and group-support team interactions pre, post and during missions. Could be used to study means of anticipating and mitigating behavioral problems within teams during expeditions.
Teamwork and social dynamics	Subscales of the Group Environment Scale and the Work Environment Scale (Moos, 2002; Moos and Insel, 2008)	These scales ask subjects to rate items such as leader support, task orientation, managerial control, and work pressure, on a periodic basis (e.g., weekly)	Used on the ISS (Kanas and Manzey, 2008)	As above

### Computerized cognitive testing

Cognitive performance of astronauts is essential for maintaining the capacity to problem solve in new situations and respond quickly in times of equipment malfunction or injury during missions. Computerized cognitive tests are based on neurophysiological measures rather than self-report, and therefore provide more objective information. In this section, we list some of the main cognitive tasks used or potentially relevant for use in space and space analogs, with particular attention to testing visuo-spatial navigation skills—a relatively new area that is relevant in caves and spaceflight and lends itself well to computerized testing. Questionnaires and ratings can also be adapted for use on a digital device such as a tablet or phone (e.g., Betella and Verschure, 2016), which may be logistically simpler than paper-based methods where they are needed in conjunction with planned computerized testing.

Reaction time and response accuracy are frequently collected in spaceflight-related cognitive testing since these measures examine crew members' abilities to react well in critical situations, and show different sensitivities to spaceflight conditions. A study on the mental performance during short term and long term space flight showed that reaction time and spatial memory were not decreased during space flight but visuo-motor tracking and dual-task capabilities were decreased (Manzey and Lorenz, 1998). Other studies looking at the single-task reaction time, visuo-motor tracking, and dual-task abilities of astronauts before and during their experience at the ISS show that all three are impaired during spaceflight (Bock et al., 2010). It has been suggested that the changes on visuo-motor tracking are a result of the microgravity effects on sensorimotor processes during spaceflight (Bock et al., 1992) and that the deficits in single- and dual-task reaction time are a result of stress and fatigue of the mission (Santy et al., 1988).

The *Psychomotor Vigilance Test (PVT)* measures how quickly the participant can respond to a visual stimulus. This test has been used on the ISS to measure behavioral and cognitive changes in astronauts' attention states, alertness, problem-solving skills, and impulsivity, and during the Mars500 mission (subjects were shown to have high levels of psychomotor vigilance performance throughout the mission Basner et al., 2014b).

The *Stroop Test* is a computerized test that can assess the attentional control of a subject by asking them to suppress irrelevant information (Stroop, 1992). A version of this test asks the subject to answer with the color of the word they are looking at and not the color that is the word actually reads. One study looked at 3 crew members during their 11 day spaceflight and found that their ability to suppress irrelevant information decreased as compared to before the mission (Pattyn et al., 2009).

Working memory is the ability to temporarily store and manipulate the information required to carry out complex cognitive tasks such as learning, reasoning, and comprehension. Both visual working memory (such as that required to store layout of a spacecraft or cave environment) and verbal working memory are important to evaluate (reviewed in Wilhelm et al., 2013). The *Sternberg memory task* asks the subject to memorize a list of words or numbers and recall whether a subsequently presented probe item had been present in the original set. Response time is measured. Several studies using this task have shown slower responses during spaceflight (Manzey et al., 1998; Kelly et al., 2005) but others have not found significant differences (Manzey et al., 1993; Newman and Lathan, 1999).

A new computerized cognitive testing battery by the name of “*Cognition*” has been developed specifically for astronauts (Basner et al., 2015) with the goal of facilitating comparison of cognitive function across analogs and spaceflight. This test covers testing on spatial orientation, emotion processing, and risk taking all encompassed in 10 neuropsychological tests. The *Spaceflight Cognitive Assessment Tool for Windows (WinSCAT)* is commonly administered to astronauts on the ISS and in spaceflight simulations (De la Torre et al., 2014), and comprises subtests measuring mathematical skills, short-term memory, working memory, attention, and spatial processing (Kane et al., 2005). A study that compared Cognition and WinSCAT showed that Cognition scores assess a variety of neurocognitive disciplines while the WinSCAT weights heavily on executive control (Moore et al., 2017). For further discussion of the measurement of working memory, attentional control, and other cognitive testing of subjects in spaceflight environments (see Strangman et al., 2014; Kanki, 2018a,b).

**Figure 4:**
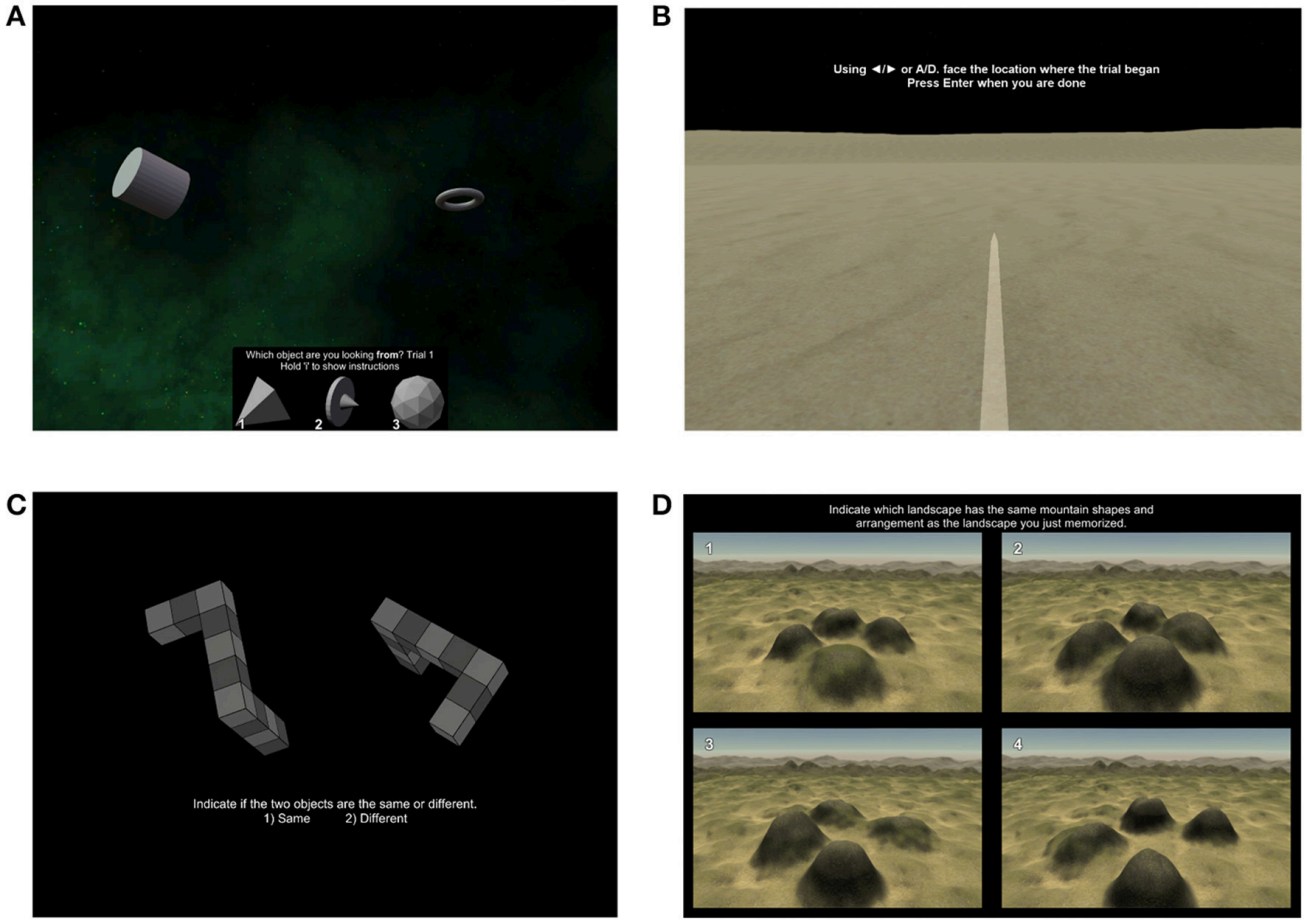
Computerized tests of visuo-spatial orientation skills. **(A)** A sample trial of the *Spatial Configuration Task*. In each trial, participants make use of landmarks in a scene to infer their location in a simple environment populated with five geometric objects. The environment (and objects' locations) remains stable throughout the test, and participants are required to build a mental representation of the locations of objects throughout the test. **(B)** A sample image of the response phase of the *Path Integration Test*. In this task, participants view two automatic first-person displacements, and must indicate the direction and distance to return to the starting point of the trial. **(C)** A sample trial of the *Mental Rotation Test* in which participants are required to mentally manipulate the objects to decide whether or not they are the same. **(D)** A depiction of the response phase of the Four *Mountains Task*. In each trial of this task, participants indicate the option which shares the same topography with the stimuli encoded immediately prior (not displayed).

Visuo-spatial orientation skills can be tested in a variety of ways that subdivide different elements of this complex phenomemon. Here, however, we highlight four tests and a questionnaire that we believe provide a comprehensive assessment of visuo-spatial orientation skills in humans. The *Spatial Configuration Test* measures the subjects' ability to create a mental representation of the environment, which is a critical process for orienting and navigating effectively in one's surroundings (Burles et al., 2017). In this test, participants view scenes from a space-like virtual environment populated with five simple, geometric objects (Figure 4A). Participants learn the positions of the objects through 60 successive trials, which are constituted by a series of first-person displacements from one object to another. At each trial, participants indicate the unseen object they are located upon. Participants' accuracy and reaction times are recorded. The *Path Integration Test* is used to estimate a subject's ability to convert visual optic flow information into a spatial representation of distances traveled in the environment (McNaughton et al., 2006). In each trial of this test, participants are presented with two displacements from within a simple environment devoid of any landmarks (Figure 4B). At the end of the two displacements, participants are asked to indicate the distance and direction to the point from which the first displacement took place (i.e., original starting point). Participants' angular and magnitude errors at each trial are recorded. The *Mental Rotation Test* characterizes a subject's ability to mentally manipulate 3-D objects in space, which is critical for the mental rotation component of spatial orientation and navigation (Kozhevnikov et al., 2006). In this test, participants view pairs of objects constructed from 10 cubes each (Figure 4C). At each of the 80 trials, participants indicate if the two objects are the same, or if they are mirror images of one another. Participants' accuracy and reaction times are recorded. The *Four Mountains Test* evaluates the ability to mentally manipulate viewpoints and recognize locations from different perspectives (Hartley et al., 2007; Hartley and Harlow, 2012). In each trial of this test, participants view a virtual scene composed of four distinct mountain peaks. After each scene, participants are required to identify the same scene, depicted from a different viewpoint, from four response options (Figure 4D). Participants' accuracy and reaction times are recorded.

The *Santa Barbara Sense of Direction Scale (SBSOD)* captures a more ecologically valid measurement of spatial orientation skills (Hegarty et al., 2002), but is still related to the aforementioned, more focused, measures of spatial abilities, and importantly, the capacity to generate mental representations of the environment (Burles, 2014). The scale consists of items evaluating an individual's subjective rating of his/her spatial orientation and navigational skills as experienced in daily life, via agreement on a 7-point scale with statements such as “I can usually remember a new route after I have traveled it only once.”

### Eye tracking and pupillometry

Eye trackers and pupillometry tools provide non-invasive and rich indices of brain function and cognition. Gaze analysis reveals attentional focus and cognition strategies (Eckstein et al., 2017), and pupil dilation reflects mental effort (Granholm et al., 1996). Previous work has used portable eye trackers by attaching video cameras onto helmets or eyeglass frames, allowing for eye movement to be recorded, modeled, and stored. To our knowledge, eye trackers have not yet been brought into cave environments (and would likely not be practical for use during exploration activities) but they have been used in a virtual cave environment (Koles and Hercegfi, 2015), and could be used to collect data pre-mission or at a base camp. Eye tracking devices have been used in parabolic flight to study visual function in microgravity (Clarke and Haslwanter, 2007). For a more detailed look on eye movements and their relation to space physiology, see the following review: (Clément and Ngo-Anh, 2013).

### Actigraphy

An actigraph is a device that can monitor movement, usually via wrist-worn accelerometers that record movements over periods of days. Some inexpensive options include those that are available commercially to consumers to track energy expenditure for fitness purposes, which have already have been used in a few studies, for example looking at physiological factors related to human performance during hiking (Divis et al., 2018). Wrist actigraphs can also provide basic information about sleep-wake cycles, and have been used in studies on sleep deprivation and sleep patterns of astronauts during spaceflight (Barger et al., 2014), and to evaluate sleep-wake circadian rhythm maintainance during Mars500 (Frey, 2013).

### Skin conductance response (SCR) and heart rate variability (HRV)

The skin conductance response (SCR) (or “galvanic skin response”) is a non-invasive measure that can examine autonomic nervous system responses such as those related to stress, emotional engagement, psychological arousal, and anticipation of decision-making outcomes. SCR is measured by placing two electrodes in contact with the skin and passing a tiny electrical charge between them; changes in electrical conductivity of the skin caused by sweat gland function are then observable (see Christopoulos et al., 2016 for a primer on SCR methods). SCR is most easily and reliably measured on the palms of the hands or soles of the feet, where the density of sweat glands that are most responsive to psychological reactions are found. Despite the development of lightweight, wearable sensors, humid cave environments and physical activity often involving the hands limit the practicality of in-cave measurement. However, other metrics such as heart rate variability (HRV; i.e., variability in heartbeat interval) can also serve as indicators for autonomic nerve responses. In a recent meta-analysis, Thayer and colleagues proposed that HRV can serve as a proxy for the integration of brain mechanisms that guide flexible behavioral control with peripheral physiology, and which can be useful for understanding stress and health (Thayer et al., 2012). HRV can be measured by electrocardiogram (ECG), which is based on recording electrical activity of the heart, or by pulse oximetry, which measures changes in reflected or transmitted light due to pulsing arterial blood. Several companies are now producing portable devices containing accelerometers, thermometers, ECG, and oximeters that can be worn attached to the body or embedded in clothing (see Hey et al., 2014 for a review of recent developments in ambulatory measurement, Antoni et al., 2017; Pinna et al., 2017 for examples of in-cave use, and Vigo et al., 2012, 2013 for reports of HRV measurements during the Mars500 project, in which evidence for autonomic changes during confinement were reported).

### Portable electroencephalography (EEG)

Electoencephalography involves non-invasively recording the electrical activity of the brain via electrodes placed on the scalp that are used to record a time series of voltage fluctuations. From EEG, researchers can learn about the strength and variability of the brain's activity within different frequency bands and across the scalp while the subject performs a task, in reaction to a stimulus, or as they rest and sleep. A variety of markers have been found for example to track workload (Coffey et al., 2012; Roy et al., 2016), drowsiness (Sahayadhas et al., 2012), and vigilance (Kamzanova et al., 2014), and have been used to study the effects of interventions such as exercise on de-conditioning during confinement (Schneider et al., 2010). It has also been used for neurofeedback, in which users learn to self-regulate brain activity (see Enriquez-Geppert et al., 2017 for a review and tutorial).

EEG has been used in caves since the 1970s (Table 4), but it was kept in an easily accessed cavern that the subject stayed in for the duration of the expedition, a situation that does not represent many contemporary exploration expeditions. Research grade EEG systems today are of higher complexity, and are still typically bulky, delicate, costly, and require special setup steps with messy electro-conductive gel. EEG equipment does exist aboard the ISS (Columbus module, launched in 2008) but does not appear to have been used extensively (De La Torre et al., 2012), for which usability issues (i.e., setup, non-wirelessness) may have been a factor (see also Clément and Ngo-Anh, 2013). Portable EEG equipment has recently been developed for specialized uses such as video gaming and in clinical diagnosis that have fewer electrodes, easier application (i.e., dry electrodes), lightweight amplifiers, and battery packs. Consumer products are more variable in quality, but as they are far less expensive and may be less susceptible to damage, and so are most suitable for expedition environments; some have been validated against lab-grade equipment on indexing neural correlates of cognition (i.e., Wang et al., 2015; Krigolson et al., 2017). New miniaturized “ear-EEG” devices that can record a subset of EEG metrics via an electrode integrated into an earplug are currently under study; once available they would further increase potential cave, analog, and spaceflight applications (Mikkelsen et al., 2015).

### Portable polysomnography (PSG)

Polysomnography consists of EEG with additional sensors for physiological information that are necessary to answer research questions about the quality and architecture of sleep (as opposed to only its timing and length, for which actigraphy is sufficient). In addition to at least once channel of EEG, PSG includes electrooculography (EOG; to detect eye movements), electromyography (EMG; to detect muscle movements and tension), and electrocardiography (ECG or an oxygen saturation sensor) to monitor heart rate. EEG systems are often developed only with waking/cognitive testing or with sleep recordings in mind, though some equipment can be used for both purposes, making possible study designs that involve some active cognitive testing before or after sleep, or for equipment sharing across experiments. In recognition that traditional PSG requires considerable expert assistance both to apply and to analyze and negatively affects the sleep of the person under observation (which is problematic for safety reasons in expedition environments), techniques such as ear-EEG (see above) in combination with automatic sleep analysis algorithms are being tested (Mikkelsen et al., 2017). Relatively inexpensive PSG headbands that are able to play the user EEG phase-locked sounds to restore fragmented sleep and improve improve memory (i.e., closed-loop auditory stimulation) have recently been validated (Arnal et al., 2017; Debellemaniere et al., 2018); these devices could be used both to record sleep data and to test the applicability of the close-loop auditory stimulation technique in expedition conditions.

### Functional near infrared spectroscopy (fNIRS)

Functional near Infrared Spectroscopy (fNIS) is a technique that is able to image correlates of brain activity based on changes in the absorption of infrared light by hemoglobin as its oxygenation state changes (reviewed in Ferrari and Quaresima, 2012). In contrast to EEG, which provides high temporal resolution about the activity of synchronized populations of neurons, fNIRs offers access to the cortical hemodynamics that have been extensively studied using fMRI. fNIRS is less sensitive to motion artifacts than either EEG or fMRI, and is somewhat portable and much less expensive than fMRI. fNIRS has been used to study a wide range of cognitive functions such as working memory, and recent developments in portability and wirelessness have extended fNIRS experiments outside of the lab (Pinti et al., 2015). The equipment and usability of fNIRS is likely not yet sufficiently developed for cave expedition use, though this area is under active development and suitable devices may become available in the near future (e.g., Klakegg et al., 2016).

## Connection to in-field study experts and cave community

Unless the researcher is adept at planning their own cave expeditions, close collaboration with researchers having field study expertise is necessary to obtain access to caves, to provide detailed knowledge of the specific cave environment (and thus minimize confounding environmental factors Brugger et al., 2018), and to support data acquisition. Speleological groups already involved in scientific work from other disciplines than human studies (including geology, hydrogeology, and biology) can help establish an appropriate underground location and a supportive environment.

The larger and more formal caving associations (e.g., International Union of Speleology, National Speleological Society of America), their publications (e.g., International Journal of Speleology) and congresses (e.g., International Speleological Congress, European Speleological Congress) are also good starting points to identify suitable groups who are motivated to support scientific work. Rescue organizations tend to be organized at the national level, and are those most concerned with health, communication, and even telemedicine underground (e.g., ICAR Alpine Emergency Medicine Commission; ICAR-MEDCOM). Among them, medical doctors and medics are good contacts through which to organize participation and data collection for conducting human research.

The recreational cave community could be of interest for recruiting enthusiastic study participants, usually for short-term data collection in a wide range of experimental conditions. Somewhat larger expeditions are organized by local and regional organizations, and tend to pool together resources from different groups to achieve specific exploration and sometimes scientific objectives. Their participants are more experienced, and their duration ranges from long weekends to several weeks. International expeditions may be funded by sponsors or institutions and include the scientific and technical experts needed to achieve a mix of exploration, documentation, and scientific objectives. They provide a heterogeneous mix of people, and complex, challenging environments (e.g., ice caves, very deep caves, very long expeditions). Finally, rescue organizations regularly train underground in simulated rescue interventions, and could offer a unique opportunity for neuroscientists particularly to study teamwork and decision-making processes in large groups under stressful conditions, as they can engage hundreds of personnel for several days (Schneider et al., 2016).

## Conclusion

Research conducted on human cognition and behavioral performance under highly challenging conditions analogous to those found in spaceflight is needed to predict and maintain high levels of human performance in future missions. It can also offer scientists who primarily work in laboratory environment unique opportunities to observe aspects of cognitive processes as they relate to real behavior under complex, realistic, extreme environmental conditions. In addition to contributing to exploration activities and fundamental knowledge of human brain function, these investigations could benefit people in safety-critical environments and occupations, such as shift-workers, firefighters, medical teams, or air traffic controllers.

Pushed by clinical research and consumer applications, devices that record neurophysiological parameters with high-fidelity non-invasively are now becoming available, making studying a wide range of topics feasible. However, particularly in neuroscience and related areas (i.e., cognition, cognitive psychology, neuropsychology), gaps must first be spanned between laboratory and field research in methods, knowledge, and scientific culture such that the advantages of expeditions as an intermediate research platform can be realized. An efficient way to ensure that high-quality research is conducted in caves and other space analogs and effectively shared is through a mutual understanding of the expedition environment, which we have outlined here, and through collaboration. As well as ensuring that the results are valid and have an impact in both space and in cognitive specializations, academic partners can arrange for the required ethical oversight, whereas partners familiar with expeditions can contribute essential knowledge to make research possible and ensure it is well conducted in the field and practically relevant. We hope that this work will support productive collaborations that extend mainstream neuroscience into unique environments and situations, to increase our understanding of real-world cognition and improve human performance and safety in operational environments. The time is ripe for neuroscience to leave the lab.

## Author contributions

LZ conducted the original literature review, as part of a systematic review on studies human physiology (in preparation) wherein neuroscience was considered a subtopic. EC and LB developed the concept for and structure of this review and prospective. NM continued the literature search and review work, read and summarized all records, and prepared the tools and equipment sections. GI and FB contributed to the sections on computerized and survey-based testing. GS and LB contributed to the sections on space analogs and connecting to the space community. NM and EC wrote the paper, with contributions and input from LZ, LB, GI, and GS.

### Conflict of interest statement

The authors declare that the research was conducted in the absence of any commercial or financial relationships that could be construed as a potential conflict of interest.

## Abbreviations

EEG: electroencephalography
EMG: electromyography
EOG: electrooculography
ESA: European Space Agency
fNIRS: functional near infrared spectroscopy
fMRI: functional magnetic resonance imaging
HRV: heart rate variability
ISS: International Space Station
NASA: National Aeronautics and Space Administration (US)
PSG: polysomnography
SCR: Skin conductance response.
